# Alzheimer’s disease histopathology in the human Down syndrome retina and in hiPSC‐derived retinal organoids

**DOI:** 10.1002/alz.093529

**Published:** 2025-01-03

**Authors:** M. Natalia Vergara, Ethan James, Michael Ha, Citlali Aguilera‐Rico

**Affiliations:** ^1^ University of Colorado Alzheimer’s and Cognition Center, and the Linda Crnic Institute for Down Syndrome, CellSight Ocular Stem Cell and Regeneration Program, Sue Anschutz‐Rodgers Eye Center, University of Colorado School of Medicine, Aurora, CO USA; ^2^ CellSight Ocular Stem Cell and Regeneration Program, Sue Anschutz‐Rodgers Eye Center, University of Colorado Anschutz Medical Campus, Aurora, CO USA

## Abstract

**Background:**

Down Syndrome (DS) is a genetic disorder caused by the triplication of human chromosome 21 that affects approximately 1 in 700 people born in the U.S. People with DS are at a greater risk of developing Alzheimer’s disease (AD), with practically all individuals developing AD histopathology and more than half progressing to dementia. However, the histopathological manifestations of AD in the DS retina have not yet been investigated. Closing this knowledge gap could lead to improved AD diagnosis and treatment for individuals with DS, since retinal structural and functional features are increasingly being explored as biomarkers of AD. In this study, we evaluated the presence of AD histopathology in human cadaver retinas from donors with DS as well as disomic controls. Moreover, we developed the first retinal organoid (RO) models of DS using patient‐derived induced pluripotent stem cells (iPSC) and evaluated their ability to recapitulate AD histopathology.

**Method:**

De‐identified human cadaver eyes from individuals with DS and disomic controls were obtained from the Miracles in Sight Eye Bank, fixed, embedded in paraffin or OCT, and sectioned for histopathological analysis. Human iPSC‐derived ROs were generated from disomic controls and donors with DS using the Zhong et al. (2014) protocol and collected at 100 days of differentiation. AD histopathology was assessed by immunoblotting and immunofluorescence staining, including retinal cell type‐specific markers and amyloid‐β (Aβ), phosphorylated Tau (pTau), and NIAD‐4 staining for amyloid plaques.

**Result:**

Histopathological analysis of human cadaveric donor retinas indicated an increased deposition of Aβ plaques and pTau in retinas from individuals with DS compared to control donor retinas, consistent with the pathology observed in the brain. DS‐ROs developed a full component of neural retinal cell types and exhibited elevated levels of Aβ plaques and pTau compared to control ROs, mirroring the phenotype observed in the native DS retina.

**Conclusion:**

AD histopathology can be observed in the human DS retina and in stem cell‐derived RO models of DS. These DS‐RO models may open new opportunities for drug screening and biomarker discovery, as well as enable comparative pathophysiological studies between DS‐ROs and the recently developed AD‐ROs.

